# Introducing a feedback training system for guided home rehabilitation

**DOI:** 10.1186/1743-0003-7-2

**Published:** 2010-01-15

**Authors:** Fabian Kohler, Thomas Schmitz-Rode, Catherine Disselhorst-Klug

**Affiliations:** 1 Dept of Rehabilitation- and Prevention Engineering, Institute of Applied Medical Engineering, RWTH Aachen University, Helmholtz Institute, Pauwelsstr 20, Aachen, 52074, Germany

## Abstract

As the number of people requiring orthopaedic intervention is growing, individualized physiotherapeutic rehabilitation and adequate postoperative care becomes increasingly relevant. The chances of improvement in the patients condition is directly related to the performance and consistency of the physiotherapeutic exercises.

In this paper a smart, cost-effective and easy to use Feedback Training System for home rehabilitation based on standard resistive elements is introduced. This ensures high accuracy of the exercises performed and offers guidance and control to the patient by offering direct feedback about the performance of the movements.

46 patients were recruited and performed standard physiotherapeutic training to evaluate the system. The results show a significant increase in the patient's ability to reproduce even simple physiotherapeutic exercises when being supported by the Feedback Training System. Thus physiotherapeutic training can be extended into the home environment whilst ensuring a high quality of training.

## Introduction

Medical rehabilitation and postoperative care is focused on restoring body or organ functions with physiotherapeutic and ergotherapeutic methods. The addressed patients require adequate and individualized therapy according to their needs to improve the chances of continuing to live independently and to quickly regain a good and efficient quality of life [1]. Medical rehabilitation is usually done in a hospital setting but to an increasing degree ambulatory [2-5].

Physiotherapy is the main rehabilitation method for a great variety of movement disorders or neurogenic dysfunctions. Examples for physiotherapy on neurogene basis is the treatment of stroke patients according to the concepts of Bobath or Vojta, PNF, motor relearning and many more [6]. Through training of everyday movements applying different training methods the neuroplasticity of the brain is used and leads to improvements in the movement capabilities of patients [7,8]. Another very important field of rehabilitation, which will be addressed in this paper, is the physiotherapeutic training for patients with skeletal dysfunctions such as bone fractures and joint replacement and also muscular, tissue or tendon disorders like impingement syndromes. Additionally a growing group of people require orthopaedic intervention and therefore physiotherapeutic training. The assessed methods are individualized and used to reduce pain, regain range of motion, stabilize joints and train harmonic movement coordination patterns and, if necessary, increase muscle strength. The goal is to enable the patient to move painlessly and harmonic in every-day situations.

The general charge for the therapist is to diagnose the movement deficits and develop an individualized physiotherapeutic training program. He then teaches these exercises to the patient. The therapist observes and controls the rehabilitation process and provides additional advice if necessary. The accuracy of exercise performance in physiotherapy in-fluences the healing process of the patient greatly. Success is deriving from form, amount and the consistency of training. In reality, the limited personal resources do not allow the accomplishment of the theoretical goals in rehabilitation.

An effective way which provides guidance and control to the patient and helps monitoring the therapy progress must be addressed to support physiotherapists in this healthcare situation. One way of supporting the healing process is using effective assistive training systems that help the patient to regain his movement capabilities [7]. These systems cannot replace the direct human interaction between therapist and patient [9] but can aid valuable support to the rehabilitation process, for both muscular-skeletal and neurogene training. A great variety of such assistive systems have been developed so far. To intensify gait rehabilitation, therapy based on treadmills was introduced in the early 1990s [10,11] and developed further by introducing exoskeleton devices [12-14] or end-effector-based systems that allow movements in the not controlled joints [15,16]. Similar development took place for the rehabilitation of upper extremities. Severely affected patients were treated by intensifying the use of the affected limb [17,18]. The Massachusetts Institute of Technology (MIT) developed a robot arm to train shoulder-elbow-movements [19-21]. Also bilateral approaches are discussed [22] with rope-kinematic robots that move patients like marionettes [23] or with two robot arms [24,25]. Another training method utilizes passive training aids [26] or passive exoskeletons [27]. The therapeutic effect of the mentioned assistive devices is still subject to discussion, but it is believed that they allow an intensification of the therapy [28-30].

The above mentioned solutions provide guidance and control for the patient, but are very expensive and need complex machinery. Furthermore, movements trained with these systems are often not self motivated but externally channelled and routed. The usage of simple training aids like isokinets, barbells, resistive elements, balls or comparable training devices create a better possibility for self-motivated training. They are easy to use, mobile and allow repetitive training but lack guidance and control. Using them in without guidance might lead to a false training and a decreasing chance of a fast recovery for the patient.

Ideally exercises should be done several times a day [31]. Extending the physiotherapeutic training to the personal environment could solve the dilemma between the burden on physiotherapeutic institutions due to the rising demand and the need of individualised frequent training. It would be a great improvement if physiotherapeutic exercise could also be performed in a home environment. This meant less ambulant consultations and less guidance by physiotherapists. The responsibility and control of the rehabilitation training is handed over from the therapist to the patient. An inexpensive and easy to use system is necessary to support the patient in his training effort, so that a controlled indirectly supervised training becomes possible.

The so far mentioned assistive devices like treadmills or exoskeleton devices provide guidance and control but are too expensive and too complex and therefore not suitable for home rehabilitation training. This is true for many other approaches as well [32-36].

We therefore aimed to develop an easy to use, cheap and mobile training system that allows home training and provides sufficient guidance and control to the patient. In this paper a smart user-tailored Feedback Training System (*FTS*) for patients in their home and work environment will be introduced. The integration and further development of the cost effective training system requires 1.) low cost training apparatus and 2.) control aspects. The latter involves a continuous feedback for the user about his performance and the possibility of tele-monitoring his efforts by healthcare professionals [37].

## Methods

### Conception

**Figure 1:**
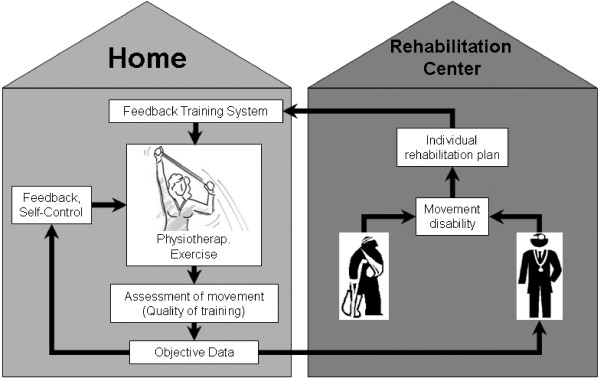
**Concept of Home Rehabilitation**.

The introduced Feedback Training System for home rehabilitation should enable the patient to perform his rehabilitation exercises on his own responsibility but controlled at home. Analogue to classic rehabilitation, the physiotherapist assesses the individual needs of the patient and defines appropriate training exercises and a resulting training plan. The exercises are then trained together with the patient. In this phase, the patients movements are supervised by the therapist and simultaneously recorded with the *FTS *to serve as reference. For each exercise a reference movement is chosen from the recorded training and stored together with the training plan in the *FTS*. In the self dependent training situation at home the system is attached to the private PC and presents information about the exercise that has to be performed according to the training plan. The training movements are being assessed quantitatively and compared to the reference movements that were defined previously. If necessary, adequate visual feedback is displayed on the computer screen to help the patient to identify possible variances in his movements and helping him to correct them (Figure 1) [38]. The assessed quantitative data should also be stored or transmitted to the therapist for later review [39]. In the end the goal must be ensuring a training of the desired movement patterns and enabling the patient to transfer these patterns into daily activities [40].

### The Feedback Training System

The Feedback Training System is based on resistive elements like gymnastic bands or tubes. They are cheap, easy to use and allow resistive training at home. To characterize a physiotherapeutic exercise, the movement path, amplitude and speed of the extremities must be assessed. Since the moved extremities lengthen the resistive element, the resulting force within the element is proportional to the amplitude and range of motion. The range of motion can therefore be estimated by measuring the force of the resistive element with an adequate force sensor.

#### Resistive Elements

The mechanical characteristics of resistive elements are similar to the ones of rubber as they are mostly derived from latex or natural rubber. The stress-strain-curve was measured to define the relation between force and elongation. The measurements were undertaken according to DIN 53504 and ISO 527-1 with a shoulder test bar S2 which is appropriate for elastomeres and natural rubber. The non-linear behaviour of the resistive elements must be considered when mathematically describing the resistive elements. Reasonable training resistances in physiotherapy lie between 10 to 40 Newton. The length of the element has to be defined by the therapist to match the boundary conditions of movement range and resulting force. With the defined length of the element, the elongation can be calculated from measured force values.

#### Force Sensor

**Figure 2:**
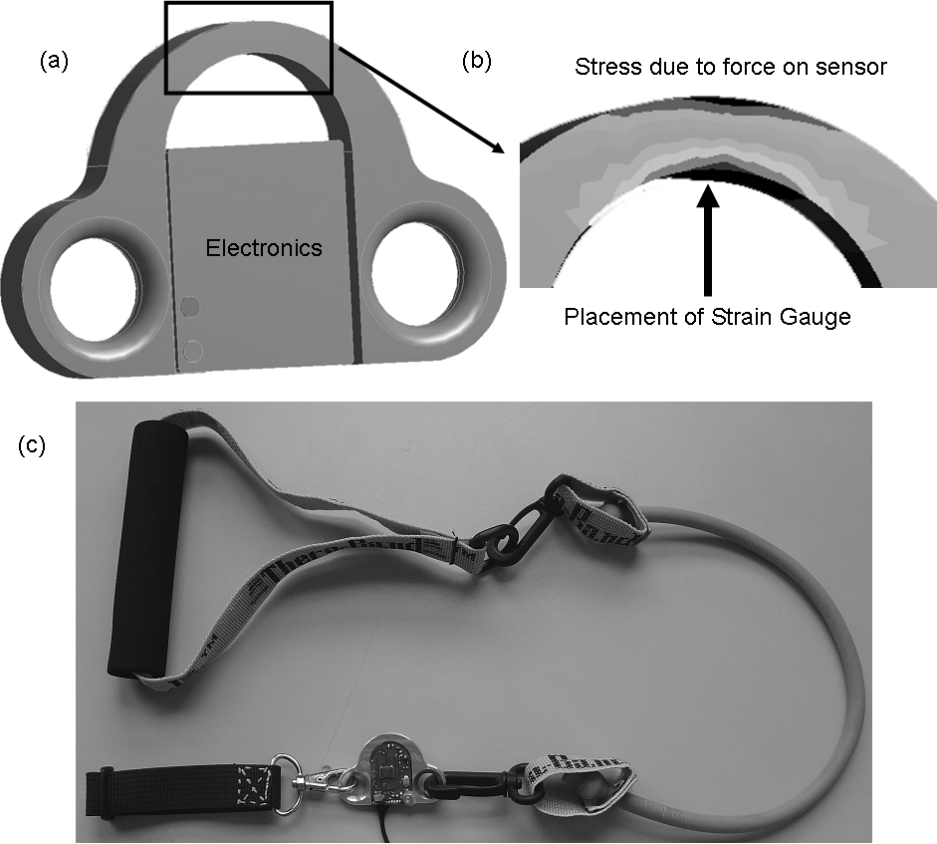
**Sensor Design: (a) Geometry of the force sensor**. (b) Stressed area when force is applied to the sensor and placement of strain gauge. (c) Final sensor with resistive element and handle.

Since the relation between force and elongation of the used resistive elements is known, the assessment of the one-dimensional force, produced by pulling the resistive element, allows the calculation of the amplitude of the movement. A sensor was developed to measure forces up to 50N with an even higher breaking stability. It has to be small and easy to attach between the resistive element and a handhold. The design shown in Figure 2a was chosen and optimized for the usual forces of physiotherapeutic training.

Figure 2b shows the stressed areas in the upper part of the U-shaped aluminium element, when a force is applied to the sensor. On this location of greatest stress a resistance strain gauge from *Vishay *[41] is applied to measure the bending of the material as a consequence of an applied force. Strain gauges change their electrical resistance with mechanical deformation, especially elongation. The maximum relative lengthening *ε *of the used strain gauge is around 0.1%.

The *K*-factor for the used strain gauges is 5, therefore the maximum change in resistance is expected to be around 0.5%. To achieve best possible results in measuring such small changes in resistance, the strain gauge is connected to a *PicoStrain PS02 *microchip from *Acam *[42]. It measures the changes of resistance in the strains by discharging a capacitor and measuring time. A second strain gauge is placed on the inner side of the aluminium sensor, where the material is minimally bent. It serves for reference temperature measurements. Each acquisition is sampled with 12bit resolution and takes about 60 *μ*s. 300 measurements are averaged for one actual value. The result is digitally transported by a SPI interface to a *Atmega 64 *microprocessor [43], which controls the the PS02-Chip and sends the data via USB to a PC.

Common rehabilitation movements with gymnastic bands last about 4 to 5 seconds (0.2 *Hz *- 0.25 *Hz*). The highest reasonable frequencies in visual feedback tasks are about 2 Hz [44-46]. Errors in slow movements (>500 ms) can be corrected directly using visual feedback, especially if the feedback is expected [47]. A flicker-free visualisation of the feedback can be achieved with frequencies of 25 Hz or greater. Therefore the acquisition rate of the whole system is set to 25 Hz.

Figure 2c shows the handles, the U-shaped aluminium sensor with included electronic and the resistive element of the final configuration. In the training situation at home, the sensor can be connected via USB with any standard PC.

#### Feedback

**Figure 3:**
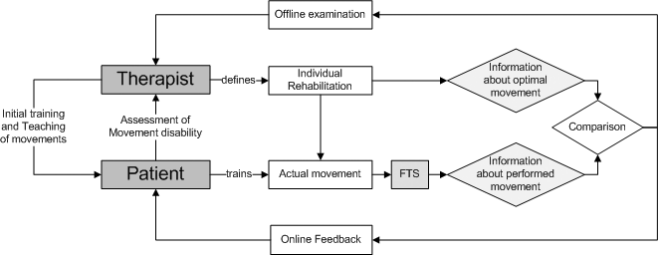
**Concept of feedback generation based on measured force data**.

The recorded data representing the performed movement must be presented with an adequate visual feedback to the patient to allow him to correct errors and to move accordingly to the individually specified training plan [48-50]. The PC screen is used to display the visual feedback. The given task and the corresponding feedback must be linked to the clearly defined functional goal: The regaining of range of motion and with it self-dependent living to encourage patients to endure in the feedback task [51]. The feedback control problem must be designed in such a way that the patient is not overburdened [52,51]. The implementation takes this into account by presenting an easy-to-follow online and direct one-dimensional feedback of the force path (Figure 3). The recorded data are additionally stored and can be examined off-line by the therapist to monitor the rehabilitation progress and interact by changing the training plan or give additional instructions to the patient if necessary.

Every rehabilitation exercise with gymnastic bands shows a characteristic path according to the strength curve, which is measured with the force sensor. Based on this path, the feedback is presented. The force path can be freely defined according to the wished movement. A common rehabilitation movement is the slow and steady stretching and releasing of the gymnastic band with predefined maximum and number of repetitions. The movement is designed in a harmonic way, since every day movements are usually harmonic and reproduced movements tend to have a bias toward harmonic movements [53,44]. Each repetition lasts usually about 4-6 seconds and is rather slow compared to more rapid preprogrammed movements [54-56]. Thus the patients should be able to use the direct feedback to increase the quality of their movements [57,47,48]. The movement pattern allows a certain tolerance from the pre-set movement path. The width *b *of the corridor is individually adapted to the patient by the physiotherapist. If the performed exercise is within the corridor, the movements can be considered to be exact enough to fulfil the therapy needs.

**Figure 4:**
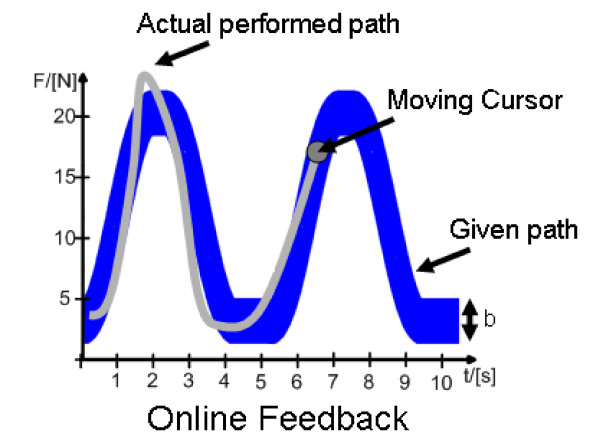
**Visual Online Feedback: Visual Feedback of the given force path of two repetitions with 5 seconds per movement, a maximum amplitude of 20N and an allowed corridor of the width *b***. The moving Cursor represents the actual force and its path is displayed as well.

The feedback is presented as an oscilloscope-like visualisation (Figure 4). The user sees the given force path and can anticipate its progression over time including amplitude, path, speed and number of repetitions. The resulting force of the actual movement is presented as a moving cursor that draws a path on the screen, while the user pursues his training movements. By comparing the given forth path with the actual performed one the user can identify errors and correct them.

This kind of feedback contributes to the learning curve, as it helps the patient to evaluate his performance and update his movement schema in case of errors [58,49]. In Figure 4 for example the subject can identify an overshoot in the first shown movement repetition. For the next repetition, he can adapt the movement amplitude to fit within the given path.

### Mathematical parameters to evaluate training movements

The performed rehabilitation movements are compared with the corresponding ideal movement that was predetermined by a therapist. The comparison is done with a set of five parameters. Each parameter was chosen to indicate the quality of the reproduced movements. If the training movements can be reproduced accurately, it can be assumed that the rehabilitation training would benefit from using the introduced Feedback Training System.

To each training exercise with resistive elements belongs an optimal strength path *y*(*t*). *x*_*i*_(*t*) represents the information about the *i*th repetition of the actual performed force path. Each repetition *x*_*i*_(*t*) consists of *M*_*i *_recorded data points. Each training exercise is trained as a set with *N *repetitions. Sets of different training exercises form a training plan.

The first parameter that was used to determine the differences of the actual forces of the subjects compared to the predetermined ones was the cross correlation coefficient. It is a measure for the reproducibility of a movement and gives an idea of the similarity of two signals. Since cross-correlations are sensitive to timing errors [53], the curves were shifted until the best fit was achieved. This also eliminated any possible delays. The cross correlation coefficient is calculated for each repetition of the recorded movement. The resulting values were averaged over the N repetitions to achieve one measure for the whole training set. The coefficient is *1 *if the performed movements are an exact copy of the given one and reaches the value *0 *if the performed movement fulfils the condition of orthogonality.

The second parameter reflects if the subject reaches the predetermined maximum amplitude of the force, respectively the range of motion and is therefore called the "Relative Amplitude Error". For each of the N repetitions the locale maximum is determined and the difference to the given amplitude is calculated. The amplitude error is normalized to the given amplitude. A value of 0 would be achieved, when the amplitude of the movement matches exactly the pre-set amplitude.

The third parameter gives an idea about the relative duration error. It compares the length of the actual movement to the given movement. The parameter is averaged over the *N *repetitions of one movement set.

The forth parameter calculates the percentage of the movement outside of the allowed movement corridor with the width *b *and is called the "Outside Parameter". While the cross correlation coefficient reflects also small variations from the given movement, the outside parameter only takes variations into account, where the movement exceeds the limitation given by the corridor. The corridor width *b *is given as a percentage of the maximum desired amplitude and allows variations of ·*b *in positive and negative direction of the exact path. The parameter for the whole training set is then calculated by equation 3.3.1.(1)

The outside parameter would indicate a perfect result for movements that are within the given corridor but are overlaid with a tremor for example. Since the movement should be smooth and steady, a fifth parameter is introduced to calculate the smoothness of the movement. Smoothness is defined as the average absolute curvature of the movement performed. Since the *M*_*i *_data points of the recorded force *x*(*t*) are equally spaced, the curvature of repetition *i *is calculated as shown in equation 3.3.2. Curvature and smoothness are parameters usually used to describe mathematic functions and have no unit.(2)

The smoothness for one repetition *i *is the average absolute value of the curvature and is then averaged for each of the *N *repetitions (3.3.3).(3)

## Evaluation

For a proof of concept and to strengthen the hypothesis that users benefit from visual feedback in the attempt to reproduce the rehabilitation movements defined by a physiotherapist, the *FTS *was evaluated in a study with 46 young and healthy subjects. The study was approved by the ethical committee of the medical faculty of the RWTH Aachen University. The subjects were divided randomly into two groups. The first group consists of 10 men (26.8 ± 5.3 years) and 6 women (26.7 ± 4.5 years) and received no visual feedback from the *FTS*. The second group consists of 10 men (27.6 ± 4.7 years) and 20 women (25.1 ± 6.3 years) and received visual feedback. If the results of the study are encouraging, further investigations with elderly and patients with movement disorders can be made.

### Method

**Figure 5:**
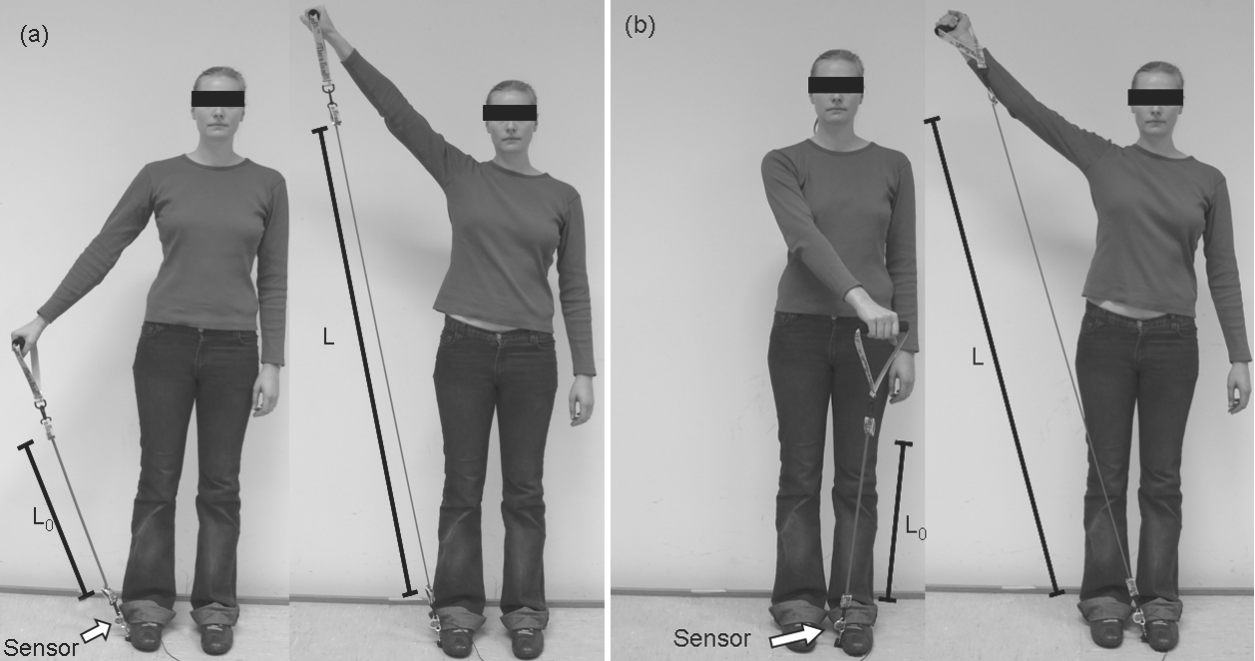
**Movement Patterns: (a) Abduction-Adduction of the right arm and (b) diagonal PNF Pattern of the right arm**.

All subjects were right handed and held the handle of the training device with the right hand and pulled against resistance while the other end was connected to the foot (Figure 5). The occurring forces were between 18N and 24N for all subjects. For each subject it was decided randomly if a either an abduction/adduction movement or a diagonal PNF pattern should be performed. All subjects were measured in 2 sets of 12 repetitions. The abduction/adduction movement begins with a horizontally extended arm and with dextrally rotated hand. The arm is then elevated and moved circularly around the shoulder joint above the head. The PNF diagonal begins with sinistral rotated stretched out arm that is held proximal in front of the body. Then the arm is moved diagonal to a distal position over the head on the right side while performing a supination in the elbow at the same time, what leads to a dextral Orientation of the hand (Figure 5). The movement patterns were taught directly prior to the measurements. Both groups were treated in the exact same way. The only difference was that one group was provided with additional visual feedback (Feedback-Group) and the other group had to perform without visual feedback (Control-Group).

The subjects performed the movements in two sets with 12 repetitions leading to 1104 different movement repetitions, 720 with visual feedback and 384 without. The movements were examined with the parameters as mentioned before. Since all parameters were calculated relative to the pre-set amplitude and given duration, the results for the two movements, Abduction/Adduction and diagonal PNF pattern were combined to compare both groups. The aim of this study was to evaluate the Feedback Training System in view of quality of rehabilitation training movements and benefit from the provided feedback. The effects are being investigated through the mentioned mathematical parameters calculated from the measured force values.

For all parameters, the mean values as well as the variances were calculated. For evaluating the differences in the parameters among different groups, analysis of variance (double-sided T-TEST with unbalanced variances) was used and calculated with *EXCEL*. Differences with *p *< 5·10^-5 ^were considered to be statistically significant.

### Results

**Figure 6:**
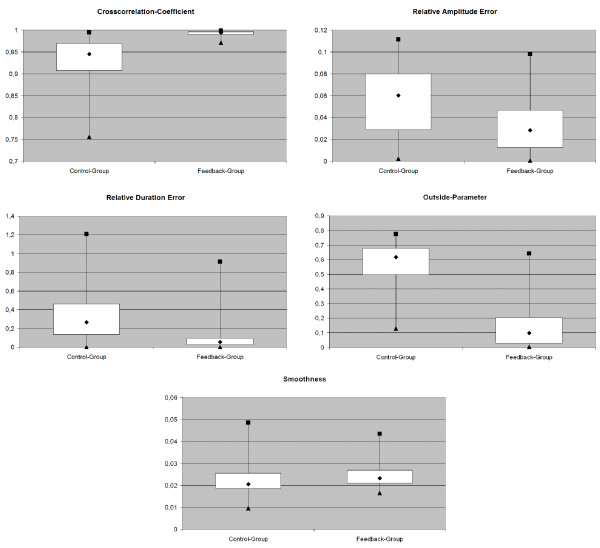
**Results for the investigated Parameters: Box Plots for Cross Correlation Coefficient, Relative Amplitude Error, Relative Duration Error, Outside Parameter and Smoothness Parameter**. Each displayed with median, 25% and 75% percentiles as well as minimum and maximum values.

Figure 6 shows the results for the investigated parameters. All parameters were plotted with *EXCEL *as box plots with minimum, maximum and median value as well as 25 and 75 percentiles.

On the basis of the recorded force data, the *Cross Correlation Coefficient *was calculated for each movement repetition. The reproducibility was then determined with a mean value of 0.93 ± 0.06 for the Control-Group and 0.99 ± 0.01 for the Feedback-Group. The differences were significantly different with a p-value of 1.2·10^-9 ^(Figure 6). The results regarding the correlation between the given ideal movement and the actually performed movements were significantly better in the Feedback-Group than in the Control-Group. The about 10 times smaller standard deviation underlines this impression. This implies that the feedback significantly improves the capability of the subjects to reproduce the given force path.

The *Relative Amplitude Error *is significantly smaller in the Feedback-Group (0.03 ± 0.03) than in the Control-Group (0.06 ± 0.03) with a p-value of 7.6·10^-7^. This proves that besides the form of the force path also the amplitude of the force and with it the desired range of motion could be reproduced more accurately than in the Control-Group. As absolute errors are used, the information if the amplitude was over- or understepped cannot be derived. If the actual movement is compared to the sharp optimal and given force path without the allowed movement corridor, it can be found that the Control-Group pulled 87.5% of the time too hard and 12.5% not hard enough while the Feedback-Group overstepped the given amplitude 58.3% and understepped it 41.7% of the time. The results of the amplitude variation are astonishing regarding the allowed movement corridor. The actually achieved variance is smaller than the allowed variance of ·*b *= 5% in each direction.

The relative duration error of the Feedback-Group (0.09 ± 0.13) was significantly smaller than for the Control-Group (0.33 ± 0.26) with a p-value of *p *= 2.22·10^-17 ^(Figure 6). The subjects of the Control-Group seemed to have fallen into an individual movement speed and maintained that speed quite steady, what is reflected in the small standard deviation of 0.26. Since the duration error only displays the absolute difference between the duration of the actual movement and the optimal movement, the duration error was further investigated to answer the question if the duration was over- or understepped within the groups. It was found that compared to the sharp optimal movement time the mean duration of the Control-Group movements were 85.4% of all repetitions too long and 14.6% the movement was to short. The Feedback-Group repetitions were 78.3% too long and 21.7% too short.

For the Control-Group the *Outside Parameter *was calculated with 0.57 ± 0.16 and for the Feedback-Group with 0.15 ± 0.15. The p-value approved statistical differences with *p *= 5.96·10^-25 ^(Figure 6). The parameter embraces the above mentioned parameters *Cross Correlation Coefficient, Relative Amplitude Error *and *Relative Duration Error *since it is sensible for movements that lie outside of the allowed force corridor around the optimal force path. It is therefore not surprising that also the *Outside Parameter *states a significant advancement for the Feedback-Group.

For both groups the *Smoothness Parameter *was calculated with 0.02 ± 0.01. The T-Test showed no significant changes with a p-value of *p *= 0.24. The *Smoothness Parameter *provides information if the feedback task changes the smoothness and steadiness of movements compared to free movements. It allows an estimation of how unsteady and turbulent the movement was performed and if these movement characteristics were negatively influenced by the visual feedback. Since the parameter shows no statistical changes between the two groups, it can be suggested that the visual feedback task did not have any negative influence on the performed movement.

## Discussion

The combined results showed evidence that the presented feedback of the *FTS *improves the capability of the subjects to reproduce given force paths reflecting the boundary conditions of form, amplitude and duration while maintaining the individual smoothness and steadiness of the movement. Even simple movements like the presented abduction/adduction and the diagonal PNF pattern of the arm benefit significantly from the provided feedback. This supports the idea of improving the quality of home rehabilitation training with the introduced system.

These results indicate that the movement speeds are well within the acceptable range of direct optical feedback [47,59,60]. The mental representation of the movements can be trained further to a higher accuracy [61,58,49]. This is emphasized by the fact that the given movement pattern does not change and the frequency is constant [44].

Since all movements were overseen by an investigator, it can be resumed that no major movement error occurred during the tests, though it is imaginable that subjects perform wrong movements while exercising with visual feedback. For example, the *FTS *in the presented form cannot distinguish between a flexion or abduction movement. Since a patient has a clear will to recover as soon as possible it can be assumed that the subjects are cooperative and want to perform the given physiotherapeutic movements in the best possible way. It can also be assumed that many wrong movements make it impossible for the patient to achieve the pre-set force paths and amplitudes, what would also be indicated by bad training results.

It was demonstrated by Todor and Cisneros that the principle difference of handedness lies in the ability to accommodate greater precision demands [57]. It must therefore be expected that the results regarding the reproduction of given physiotherapeutic movement paths for the weak side might be not as good in contrast to the strong side. Learning phases might also be longer to achieve the same results compared to the strong side.

The introduced Feedback Training System can also be extended with other additional sensors like the use of web cams, accelerometers, gyroscopes or magnetometers to aid more information to the feedback data basis [62].

The *FTS *fulfils the requirements of a small, cheap and easy to use training device for physiotherapeutic exercises at home. By supporting their efforts with adequate online feedback, it supports the patient with guidance and control, so he can perform the predefined movements with high accuracy. The *FTS *seems to be a promising way to support physiotherapeutic training at home. The results encourage an investigation of the practicability of the system with elderly patients that are affected by movement disorders in the upper extremities.

## Conclusion

A Feedback Training System has been introduced that allows home rehabilitation with resistive elements and provides the patient with guidance and control. It is cost effective, movable, easy to use and assures a higher quality of movements performed in comparison to an uncontrolled unguided home rehabilitation.

## Competing interests

The authors declare that they have no competing interests.

## Authors' contributions

FK developed the training system, designed and carried out the study and the statistical analysis and wrote the manuscript. TSR gave valuable feedback and expert guidance throughout this study and manuscript writing. CDK participated in the development of the training system and the statistical analysis, helped revising the manuscript and gave final approval to the version of the manuscript to be submitted. All authors read and approved the final manuscript.
